# Mathematical Modeling of In Vitro Rumen Fermentation Kinetics in Capiaçu Elephant Grass Silages with Inclusion of Dehydrated Cashew Pseudo-Fruit

**DOI:** 10.3390/ani15233481

**Published:** 2025-12-03

**Authors:** Isadora Osório Maciel Aguiar Freitas, Antonio Leandro Chaves Gurgel, Luís Carlos Vinhas Ítavo, Luiz Antônio Rodrigues, Vitor Cardoso Queiroz, Edy Vitoria Fonseca Martins, Marcos Jácome de Araújo, Tairon Pannunzio Dias-Silva, João Virgínio Emerenciano Neto, Alfonso Juventino Chay-Canul

**Affiliations:** 1 Professora Cinobelina Elvas Campus, Federal University of Piauí, Bom Jesus CEP 64900-000, PI, Brazil; isadora.aguiar@ufpi.edu.br (I.O.M.A.F.); vitorcardosoqueiroz@ufpi.edu.br (V.C.Q.); edy29813@gmail.com (E.V.F.M.); jacome@ufpi.edu.br (M.J.d.A.); pannunzio@ufpi.edu.br (T.P.D.-S.); 2 College of Veterinary Medicine and Animal Science, Federal University of Mato Grosso do Sul, Campo Grande CEP 79070-900, MS, Brazil; luis.itavo@ufms.br (L.C.V.Í.); luiz18mv@hotmail.com (L.A.R.); 3 Academic Unit Specialized in Agricultural Sciences, Federal University of Rio Grande do Norte, Macaiba CEP 59280-000, RN, Brazil; joao.emerenciano@ufrn.br; 4 División Académica de Ciencias Agropecuarias, Universidad Juárez Autónoma de Tabasco, Villahermosa CEP 86040, Tabasco, Mexico; alfonso.chay@ujat.mx

**Keywords:** moisture absorbers, fermentation kinetics, ensiling, in vitro gas production, non-linear functions, Richards model

## Abstract

**Simple Summary:**

The search for sustainable animal feed alternatives has encouraged the use of tropical forages and agro-industrial by-products. This study investigated the fermentation process of silages made from Capiaçu elephant grass, a forage widely used in tropical regions, with the addition of different amounts of dehydrated cashew pseudo-fruit, which is a by-product of cashew processing. To do so, mathematical models that can predict how gas is released during fermentation were compared, which reflects how well the feed can be digested by animals. Among the models tested, the Richards model was the most accurate and reliable in describing the fermentation process. It was also found that adding dehydrated cashew pseudo-fruit to the silage improved fermentation, suggesting better feed quality for ruminant animals. These findings are important as they show that a local by-product can be used to improve the nutritional value of forage, contributing to more efficient and sustainable livestock production, reducing waste from the cashew industry, and supporting food security in tropical regions.

**Abstract:**

This study aimed to evaluate and compare the performance of five mathematical models: Gompertz, Ørskov & McDonald, Brody, Richards, and the Dual Pool Logistic model, in describing the in vitro gas production kinetics of Capiaçu elephant grass (Pennisetum purpureum Schum ‘BRS Capiaçu’) silages. The effect of including dehydrated cashew pseudo-fruit on the in vitro degradation curves was also assessed. A completely randomized design was adopted, using Capiaçu silages containing 0%, 10%, 20%, or 30% dehydrated cashew pseudo-fruit. Rumen fermentation kinetics were measured through cumulative in vitro gas production. Model performance was evaluated using the Akaike Information Criterion (AIC), coefficient of determination (R^2^), concordance correlation coefficient (CCC), and mean square prediction error (MSPE). Accuracy (pMSPE) and precision (AIC) were also considered. The Richards model performed best with the lowest AIC (1119.07) and MSPE (0.246) and the highest R^2^ (0.917) and CCC (0.966). It was over 350 times more likely to provide a correct fit (*p* < 0.05) compared to the other models. Significant differences (*p* < 0.05) were observed between degradation curves as a function of the pseudo-fruit inclusion level. Increasing pseudo-fruit inclusion improved silage composition, raising total digestible nutrients (from 54.6% to 67.1%) and reducing neutral detergent fiber (from 58.5% to 42.3%), which directly enhanced fermentation kinetics. These results indicate that the Richards model is the most suitable for describing the fermentation kinetics of Capiaçu elephant grass silages. Moreover, linking model performance to practice, the Richards model provides a reliable tool for determining optimal inclusion levels of dehydrated cashew pseudo-fruit (up to 30%), supporting better silage nutritional quality and more efficient feed utilization in ruminant production systems.

## 1. Introduction

The elephant grass cultivar BRS Capiaçu (*Pennisetum purpureum* Schum. ‘BRS Capiaçu’) is notable among tropical grasses for its high yield, quick regrowth, and ability to adapt well to different soil and climate conditions [1,2]. However, the ensiling of this forage often faces challenges due to its low dry matter content (DM: 20–25%) and decreased soluble carbohydrate levels at harvest [3,4]. This affects the fermentation process and the final quality of the feed during forage shortages.

The incorporation of agro-industrial byproducts as additives in elephant grass silage represents a viable strategy for increasing the dry matter (DM) content of the silage, while also providing an additional source of soluble carbohydrates that enhance the fermentation process [5]. In this context, the inclusion of dehydrated cashew pseudo-fruit (*Anacardium occidentale*) as a moisture-sequestering additive emerges as a promising approach to increase the DM content of elephant grass silage and contribute to water-soluble carbohydrates [6], thereby minimizing losses during the process. However, when adding a byproduct to silage, it is essential to evaluate the degradation potential, as such byproducts may affect its degradability [7].

Studies on silage components and degradability are essential for determining the ideal proportion of additives, ensuring their effectiveness in both quantitative digestion and the improvement of the silage fermentation profile [8]. The in vitro cumulative gas production technique is widely used to perform gravimetric and metabolic evaluations of feeds [9,10,11]. Gas release from feed inoculated with rumen fluid serves as an indicator of microbial activity, as gas production results from fermentation processes [12]. However, it is essential to use the most appropriate mathematical model to accurately estimate fermentation parameters, ensuring a proper fit of the gas production curves, as fermentation kinetics vary depending on the feedstuffs evaluated [11]. Furthermore, it is important to note that the characteristics of these curves may vary significantly depending on the model adopted [13].

Some researchers have chosen to employ non-linear models in in vitro gas production kinetics studies, such as the Gompertz, Richards, Brody, and Ørskov and McDonald models [14]. Among these, the modified Dual Pool Logistic model proposed by Schofield et al. [15] stands out, as it has been widely used to estimate in vitro gas production of feedstuffs and diets intended for ruminant animals [10,16]. However, before selecting a model, it is essential to conduct a careful evaluation of the different available options, considering the specificities of each experimental situation [17].

In addition to classical non-linear models, the Dual Pool Logistic model was considered because it explicitly distinguishes between gas production derived from fiber and non-fiber carbohydrates [15]. This feature is particularly relevant to the present study, since dehydrated cashew pseudo-fruit is a rich source of non-fiber carbohydrates [6], and its inclusion is expected to alter the balance between the fiber and non-fiber fractions in the silage. However, to date, little is known about whether cashew pseudo-fruit effectively improves the fermentation kinetics of elephant grass silage and, if so, which mathematical model best describes these changes.

Based on this information, the present study evaluated the Gompertz, Brody, Ørskov and McDonald, Richards, and Dual Pool Logistic mathematical models to describe the in vitro gas production kinetics of BRS Capiaçu silages with the inclusion of dried cashew pseudo-fruit. In addition, the study analyzed the effects of dried cashew pseudo-fruit inclusion on the in vitro rumen degradation curves of these silages.

## 2. Materials and Methods

The experiment was conducted in the Forage and Pasture sector of the Professora Cinobelina Elvas Campus (CPCE) of the Federal University of Piauí (UFPI), located in the city of Bom Jesus, Piauí, Brazil. The city is located in the southern region of Piauí state, in the Gurguéia microregion, at a latitude of 09°04′28″ south, longitude of 44°21′31″ west, and altitude of 277 m.

A completely randomized experimental design was adopted, with four treatments and six replications. The treatments consisted of BRS Capiaçu elephant grass silages with the inclusion of dehydrated cashew pseudo-fruit at levels of 0%, 10%, 20%, and 30%, as fed, used as an additive. The grass was harvested approximately three months after regrowth and chopped into particles of approximately 2–3 cm using a stationary forage chopper. The cashew pseudo-fruit was collected from the fruit production sector of the Alvorada do Gurguéia Experimental Farm, part of CPCE/UFPI. The material was spread in an open, sunlit area and turned daily for seven days until it reached approximately 80% dry matter. The dry matter content was monitored daily by collecting samples and determining the moisture in a forced-air oven at 65 °C. Ambient temperature was also recorded throughout the drying period, with average, minimum, and maximum values of 28 °C, 22 °C, and 35 °C, respectively. After reaching the target dry matter (80%), the material was collected, homogenized, and ground in a forage chopper equipped with a 3 mm diameter sieve.

Twenty-four cylindrical PVC silos, each measuring 10 cm in diameter and 50 cm in length, were used as experimental units, with six silos assigned to each treatment. Compaction was performed using wooden rods, applying sufficient pressure to achieve a density of 600 kg/m^3^ in each experimental unit, ensuring the same force was applied to all silos. The silos were then sealed with plastic sheets secured with adhesive tape. During both the preparation process and the entire storage period, the silos were kept in a covered environment at a constant temperature of 25 °C.

After 76 days, the silos were opened, and the top five centimeters of silage were discarded. A sample was then collected from each silo to evaluate the chemical composition of the silages (Table 1). Dry matter (DM), mineral matter (MM), crude protein (CP), and ether extract (EE) contents were determined using the methods described by the AOAC [18]. Neutral detergent fiber (NDF) and acid detergent fiber (ADF) were analyzed according to the procedures described by Van Soest et al. [19]. Non-fiber carbohydrates (NFC) were estimated following the methodology proposed by Sniffen et al. [20]. Total digestible nutrient (TDN) content was determined based on NRC recommendations [21].

Cumulative gas production analysis was conducted at the Applied Nutrition Laboratory of the School of Veterinary Medicine and Animal Science, Federal University of Mato Grosso do Sul. All procedures related to animal handling and rumen fluid collection were previously approved by the Animal Use Ethics Committee of the same institution, under protocol No. 1.216/2022. A total of 500 mL of rumen fluid was collected from each of the three castrated male Nelore steers, which weighed on average 350 ± 20 kg, via rumen cannula to create a composite rumen fluid sample. The animals were adapted to the diet for 14 days prior to sampling and were fed ad libitum with a basal diet consisting of elephant grass silage supplemented with ground corn and soybean meal. Rumen fluid was collected in the morning, before the animals received fresh feed, to ensure a standardized microbial and fermentation condition. The collected material was filtered through multiple layers of gauze and stored in individually preheated thermal flasks to maintain a temperature of 39 °C.

In vitro cumulative gas production was measured using the ANKOM RF Gas Production System. Twenty-four flasks (310 mL), each equipped with a pressure sensor wirelessly connected to a computer, were used. Each flask contained triplicate samples incubated for 24 h. The flasks were filled with 0.5000 ± 0.0005 g of silage and 100 mL of a buffer solution composed of solutions A and B, as described by Tilley and Terry [22].

The mixture was preheated to 39 °C prior to the addition of 25 mL of inoculum, obtained by combining rumen fluid collected from three bovines. Before attaching the pressure sensors, CO_2_ was purged from the flasks to create an anaerobic environment. Three additional flasks containing only solutions A and B and the rumen fluid were also incubated as blanks to account for gas production from microbial activity alone. Data were recorded every 5 min and subsequently processed to calculate cumulative gas production, expressed as mL of gas per 100 mg of incubated dry matter (DM).

Cumulative gas production recorded over 24 h was fitted using five mathematical models, as described in Table 2. Biologically, the parameters of the different models can be interpreted as follows: P(t) represents the cumulative gas production at time t. Parameter “A” in the Gompertz, Brody, and Richards models represents the asymptotic gas production (mL of gas per 100 mg of DM), while in the Ørskov & McDonald model, it represents the gas production of the soluble fraction. Parameter “B” in the Gompertz, Brody, and Richards models corresponds to the particle colonization time (hours), or lag time. In the Ørskov & McDonald model, it refers to the gas production from the potentially degradable fraction, assuming sufficient incubation time. Parameter “C” in the Gompertz, Brody, and Richards models corresponds to the specific gas production rate (mL of gas per hour); in the Ørskov & McDonald model, it is the gas production rate of fraction B. Parameter “D” defines the inflection point of the curve in the Richards model.

In the Dual Pool Logistic model, “A” and “D” represent the volumes of gas produced by the degradation of non-fiber and fiber carbohydrates, respectively; “B” and “E” are the respective degradation rates of these carbohydrates; and “C” represents the particle colonization time (hours), or lag time.

Model parameters were estimated using the modified Gauss–Newton method via the NLIN procedure in SAS, Version 14 (SAS University Edition, SAS Institute Inc., Cary, NC, USA), with a maximum of 100 iterations. Due to convergence difficulties with the Gauss–Newton method, the Richards model was fitted using the Levenberg–Marquardt algorithm, which is more robust for highly non-linear models [27]. Convergence was achieved after a maximum of 300 iterations, ensuring stable and reliable parameter estimates.

The criteria used to evaluate the models included the Akaike Information Criterion (AIC), the coefficient of determination from a linear regression of predicted versus observed data (R^2^), the concordance correlation coefficient (CCC), and the mean squared prediction error (MSPE). Model accuracy was assessed by analyzing the paired mean squared prediction error (pMSPE), and precision was evaluated using AIC [18]. Statistical analyses for model evaluation and comparison were performed using the Model Evaluation System software, version 3.2.2.

Once the model that best described the average in vitro gas production curve was selected, the effect of silages on gas production kinetics was evaluated using a dummy variable [28]. A significance level of 5% was adopted for all statistical analyses.

## 3. Results

**Figure 1:**
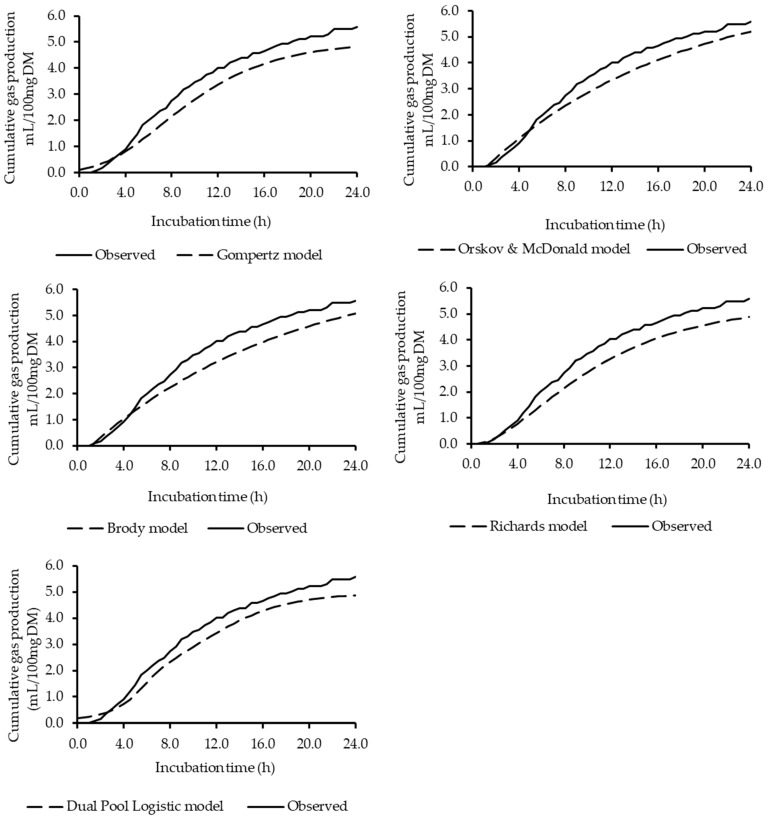
Cumulative gas production curves of BRS Capiaçu silages with the inclusion of dehydrated cashew pseudo-fruit: observed and estimated values for each fitted model.

The Richards model performed best, with the lowest AIC values, indicating a superior quality of fit, while the Ørskov & McDonald and Brody models exhibited the highest values for this criterion (Table 3). The R^2^ values from the linear regression between predicted and observed data also demonstrated greater accuracy for the Richards model. Furthermore, all models were considered accurate and precise based on the concordance correlation coefficient (CCC > 0.95), with the Richards model again showing the highest value (Table 3). The MSPE analysis supported these findings, indicating that the Richards model had the lowest magnitude of error, demonstrating a greater ability to accurately estimate gas production. Therefore, based on the evaluated criteria (AIC, R^2^, CCC, and MSPE), the Richards model most accurately described the average gas production curve of the studied silages. The cumulative gas production curves for the diets, as estimated by each model, are presented in Figure 1.

When the models were compared in terms of accuracy using the mean squared paired prediction error (MSPE), no significant differences were observed among the Gompertz, Richards, and Dual Pool Logistic models (*p* > 0.05), all of which were significantly more accurate (*p* < 0.05) than the Brody and Ørskov & McDonald models. In contrast, when model accuracy was evaluated based on the AIC, the Richards model was found to be 369.5, >10,000, >10,000, and 6782.2 times more likely to be correct (*p* < 0.05) than the Gompertz, Ørskov & McDonald, Brody, and Dual Pool Logistic models, respectively.

**Figure 2:**
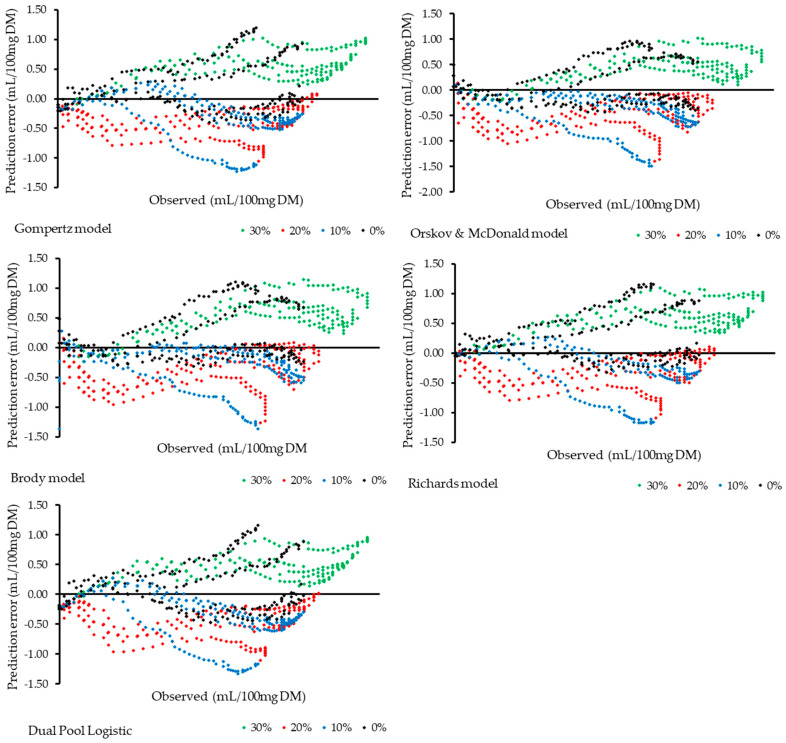
Distribution of model prediction errors as a function of observed cumulative gas production at each level of dehydrated cashew pseudo-fruit inclusion.

The distribution of prediction errors among treatments reveals systematic variations associated with the inclusion level (Figure 2). It is observed that the 0% and 30% treatments showed greater error dispersion, indicating lower model accuracy at these extremes. In contrast, the 10% and 20% treatments showed errors concentrated around zero, suggesting better predictive performance. Furthermore, it is noted that the different models exhibited distinct behaviors in both the shape and magnitude of the errors across treatments, reflecting variations in each model’s sensitivity to changes in the data.

There was a significant difference (*p* < 0.05) among the cumulative gas production curves fitted using the Richards model, according to the inclusion levels of dehydrated cashew pseudo-fruit (Table 4). Therefore, a single average (or global) model is not adequate to represent all the silages evaluated, making it necessary to fit a specific model for each inclusion level of the pseudo-fruit.

In this context, cumulative gas production curves were fitted for each silage, showing differences in the parameters estimated by the Richards model according to the levels of cashew pseudo-fruit inclusion. A higher asymptotic gas production (A) and a higher specific gas production rate were observed at the 30% inclusion level, while at the same level, the shortest colonization time (B) was recorded (Table 5).

## 4. Discussion

Several statistical techniques are available to assess the precision and accuracy of mathematical models [17,29,30]. However, none of these tools alone is capable of comprehensively evaluating model performance [17]. Even when multiple statistical methods are combined, they may still be insufficient to clearly determine which model is most efficient, particularly when the differences between models are minimal.

In this study, all tested models accurately and precisely described the in vitro gas production kinetics of the evaluated silages. Although the Richards model performed slightly better based on the statistical criteria adopted (Table 3), this advantage alone is not sufficient to justify its selection as the most appropriate model. This is due to convergence issues encountered during the iterative fitting process, which required the use of the Levenberg–Marquardt algorithm and an increased number of iterations. Similar convergence difficulties with the Richards model have been reported by other authors [14,31,32], reinforcing the need for caution in its application. This is particularly relevant when considering the principle of parsimony, which states that when two models yield similar estimates, the simpler one should be preferred [33,34].

In this context, beyond the statistical criteria commonly applied for model selection in studies describing gas production kinetics of feeds or diets [9,35,36], models were also compared in terms of accuracy and precision, as recommended by Tedeschi [17]. Predictive accuracy was evaluated using the pMSPE, which quantifies the closeness between predicted and observed values and enables direct model comparisons under the same conditions, thereby minimizing the effect of shared sources of variation [37]. Relative precision was assessed using the AIC, which, based on Kullback–Leibler information theory, measures the information loss of each model and balances goodness of fit against parsimony [38]. Thus, while pMSPE reflects predictive accuracy, AIC was interpreted as an indicator of relative precision and parsimony.

The results showed no significant differences in precision among the Gompertz, Richards, and Dual Pool Logistic models. However, regarding accuracy, the Richards model was 369.5, >10,000, >10,000, and 6782.2 times more likely to be correct than the Gompertz, Ørskov & McDonald, Brody, and Dual Pool Logistic models, respectively. Although the Richards model occasionally presented convergence challenges, its superior predictive accuracy clearly outweighed these limitations. In addition, the Richards model provides a more versatile alternative to classical three-parameter functions, such as the monomolecular, logistic, Gompertz, and Brody models. These traditional models have rigid curve shapes with fixed inflection points, restricting their applicability to diverse biological scenarios. By incorporating an additional parameter (Table 2), the Richards model allows greater flexibility in representing sigmoid curves, enabling both symmetric and asymmetric shapes. Consequently, it encompasses the three-parameter models as special cases while offering a biologically meaningful framework that adapts more effectively to data with variable curve asymmetry [26]. Together, these features justify its selection as the most appropriate model for describing the in vitro cumulative gas production kinetics of the evaluated silages.

Furthermore, the Richards model has been widely recommended for describing the average in vitro gas production curve of several feeds intended for ruminant animals. Teixeira et al. [39] evaluated the kinetic parameters of rumen degradation in protein concentrates (soybean meal and cottonseed meal) and found the Richards model to be the most appropriate. Similarly, Zornitta et al. [15] investigated the effects of combining monensin and probiotics (*Bacillus toyonensis* + *Saccharomyces boulardii*) on the degradation kinetics of corn silage and reported a better fit using the Richards model. Gurgel et al. [11] also recommended the Richards model for describing the in vitro gas production kinetics of diets containing Gliricidia hay or silage.

Despite these recurring recommendations, there are divergences in the selection of the most appropriate model [9,16,39]. This variation is theoretically justifiable, as model choice depends on the fermentation patterns of the feeds being evaluated. Therefore, in vitro gas production studies should always include careful evaluation and selection of mathematical models to ensure more robust and representative interpretations of the fermentation processes under investigation.

The choice of the most appropriate mathematical model is a crucial step in the analysis of experimental data, especially when aiming to accurately represent complex biological phenomena. The observed results show that different models respond differently to variations in treatments, exhibiting specific behaviors regarding the magnitude and distribution of prediction errors (Figure 2). This reinforces the idea that there is no universally superior model, but rather models that better fit specific experimental conditions. Using an inadequate model may lead to misinterpretations, compromising the accuracy of estimates and decision-making based on the data. Therefore, it is essential to consider the characteristics of the experiment, such as the nature of the response variable and the range of treatments, when selecting the model, in order to ensure greater robustness and reliability in the inferences made.

The differences observed in the gas production curves of Capiaçu elephant grass silages with varying levels of dehydrated cashew pseudo-fruit inclusion, as fitted by the Richards model (Table 4 and Table 5), can be directly linked to changes in the chemical composition of the silages (Table 1). The addition of dehydrated pseudo-fruit increased DM, CP, NFC, and total digestible nutrient contents, while reducing NDF and ADF concentrations.

These compositional changes suggest an improvement in the nutritional quality of the silage and help to explain the observed fermentation kinetics. The higher NFC content (41.2% at 30% inclusion) likely accelerated microbial colonization, as reflected by the shorter colonization time (B = 0.97 vs. 1.09 at 0% inclusion). Similarly, the reduction in NDF content reduced physical barriers to microbial attachment, enhancing substrate accessibility and contributing to the higher asymptotic gas production (A = 6.18 vs. 4.87 mL/100 mg DM). Thus, the increased availability of readily fermentable carbohydrates, coupled with the lower proportion of lignified fiber, supports faster microbial activity and more efficient fermentation dynamics in silages with higher levels of pseudo-fruit inclusion [40,41].

Despite the insights provided by this study on the fermentation kinetics of silages with agro-industrial byproducts and the selection of suitable models, several limitations should be acknowledged. First, the in vitro results may not fully reflect in vivo responses, as factors such as rumen passage rate and animal physiology can influence feed degradation [42]; therefore, animal studies are necessary to evaluate effects on productive performance. Second, the 24-h incubation period may not capture the complete breakdown of fiber fractions, although previous evidence indicates that, with particle sizes of approximately 1 mm, results tend to reach a plateau within this timeframe [43]. Finally, occasional convergence issues with the Richards model may limit its routine application, despite its high accuracy, highlighting the need to identify more robust models with easier convergence. Recognizing these limitations underscores areas requiring caution and further investigation in future studies.

## 5. Conclusions

The Richards model most accurately described the in vitro gas production kinetics of BRS Capiaçu elephant grass silages across different levels of dried cashew pseudo-fruit inclusion. Significant differences in rumen degradation kinetics were observed among the inclusion levels, confirming the influence of the pseudo-fruit on fermentation. Based on these findings, the inclusion of dried cashew pseudo-fruit at levels up to 30% is supported for improving fermentation kinetics.

## Figures and Tables

**Table 1:** Chemical composition of Capiaçu elephant grass silage with different levels of dehydrated cashew pseudo-fruit.

Variables (% DM)	Inclusion Level of Dehydrated Cashew Pseudo-Fruit (%)			
	0	10	20	30
Dry matter ^1^	17.7	26.4	28.2	34.4
Ash	6.6	6.4	6.1	5.7
Crude protein	4.9	6.6	7.8	8.3
Ether extract	19.4	25.5	25.8	24.4
Neutral detergent fiber	58.5	52.1	47.5	42.3
Acid detergent fiber	32.3	30.0	29.2	25.7
Non-fiber carbohydrates	27.9	32.3	35.9	41.2
Total digestible nutrients	54.6	58.9	63.0	67.1

DM: dry matter; ^1^ (g/kg as fed).

**Table 2:** Models considered in the study to describe the in vitro gas production curve of BRS Capiaçu silage with the inclusion of dehydrated cashew pseudo-fruit.

Nº	Model	Equation	Parameters	Reference
1	Gompertz	*P*(*t*) = Aexp((−Bexp(−C*t*))	3	[23]
2	Orskov & McDonald	*P*(*t*) = A + B(1 − exp(−Ct))	3	[24]
3	Brody	*P*(*t*) = A(1 − Bexp(−Ct)	3	[25]
4	Richards	*P*(*t*) = A(1 − Bexp(−C*t*))^D^	4	[26]
5	Dual Pool Logistic	*P*(*t*) = A/(1 + exp(2 − 4B(t − C))) + D/(1 + exp(2 − 4E(t − C)))	5	[15]

**Table 3:** Parameters and evaluation of the adequacy of models for estimating in vitro gas production of BRS Capiaçu silage with the inclusion of dehydrated cashew pseudo-fruit.

Nº	Model	Parameters					Model Evaluation			
		A	B	C	D	E	AIC	R^2^	CCC	MSPE
1	Gompertz	5.03 ± 0.06	3.89 ± 0.17	0.19 ± 0.001	-	-	1130.89	0.916	0.955	0.247
2	Orskov & McDonald	−0.50 ± 0.06	7.48 ± 0.22	0.06 ± 0.003	-	-	1183.57	0.910	0.953	0.270
3	Brody	6.98 ± 0.25	1 ± 0.01	0.06 ± 0.003	-	-	1183.45	0.910	0.953	0.264
4	Richards	5.36 ± 0.15	1.03 ± 0.11	0.13 ± 0.01	2.03 ± 0.52	-	1119.07	0.917	0.966	0.246
5	Dual Pool Logistic	1.20 ± 0.17	0.25 ± 0.04	3.50 ± 0.20	3.76 ± 0.15	0.07 ± 0.03	1136.71	0.915	0.965	0.253

AIC: Akaike Information Criterion; R^2^: coefficient of determination of a linear regression of predicted data on observed data; CCC: coefficient of correlation and concordance; MSPE: mean square of the prediction error.

**Table 4:** Comparison of full and reduced model fits for different inclusion levels of cashew pseudo-fruit in silage.

	10	20	30
0	<0.0001	<0.0001	<0.0001
10	-	<0.0001	<0.0001
20	-	-	<0.0001

The “full model” refers to separate fits for each inclusion level, while the “reduced model” refers to a single global fit across all inclusion levels. Reported *p*-values indicate whether model parameters differ significantly between levels. A *p*-value < 0.05 denotes a significant difference between the full and reduced models for the inclusion levels of dehydrated cashew pseudo-fruit in the corresponding row × column combinations.

**Table 5:** Richards model adjusted for in vitro gas production of BRS Capiaçu silages with the inclusion of dehydrated cashew pseudo-fruit.

Inclusion Level of Dehydrated Cashew Pseudo-Fruit (%)	Equation	RMSE	R^2^
0	*P*(*t*) = 4.87 × (1 − 1.09*exp*(−0.10 − *t*))^2.05^	0.03	0.99
10	*P*(*t*) = 4.61 × (1 − 1.06*exp*(−0.12 − *t*))^2.3^	0.10	0.99
20	*P*(*t*) = 5.66 × (1 − 1.04*exp*(−0.13 − *t*))^2.50^	0.19	0.99
30	*P*(*t*) = 6.18 × (1 − 0.97*exp*(−0.17 − *t*))^1.99^	0.04	0.99

RMSE: root mean square error; R^2^: coefficient of determination of the model.

## Data Availability

The raw data supporting the conclusions of this article will be made available by the authors upon request.
